# Trends in Women Authoring Editorials in Cardiothoracic Surgery Journals

**DOI:** 10.21470/1678-9741-2025-0395

**Published:** 2026-06-08

**Authors:** Emily Hyunmin Lee, Zoya Gomes, Jinny Minji Kim, Kiera Liblik, Nicole Travis, Susan Moffatt-Bruce, Marc Pelletier, Mohammad El-Diasty

**Affiliations:** 1 Queen’s University School of Medicine, Kingston, Ontario, Canada; 2 Faculty of Medicine, Dalhousie University, Halifax, Nova Scotia, Canada; 3 Temerty Faculty of Medicine, University of Toronto, Toronto, Ontario, Canada; 4 Department of Urology, Queen’s University, Ontario, Canada; 5 Lahey Clinic, UMass Chan Lahey Medical School, Burlington, Massachusetts, United States of America; 6 Yale School of Medicine, Yale University, New Haven, Connecticut, United States of America; 7 Cardiac Surgery Department, Harrington Heart and Vascular Institute University Hospitals, Cleveland, Ohio, United States of America

**Keywords:** Authorship, Cardiothoracic Surgery, Gender, Gender Equity.

## Abstract

**Introduction:**

Women have historically been underrepresented in leadership positions and academia in cardiothoracic surgery, creating barriers to career advancement and limiting role models for trainees. While publications are used to measure success in academia, invited articles such as editorials often represent a formal recognition of expertise. The objective of this study was to identify trends in the gender of editorial authors published in cardiothoracic surgery journals.

**Methods:**

Editorials published between 2018 and 2022 across 16 peer-reviewed cardiothoracic surgery journals were analyzed. Author gender was estimated using a validated tool (https://gender-api.com/) with additional verification using available institutional profiles.

**Results:**

In total, 806 editorials were published with a total of 1,858 authors (293 women, 16%). Women authors were predominantly from the United States of America (45%) followed by India (9%) and Germany (8%). The percentage of women first authors increased between 2018 and 2022 (P < 0.001); 9% in 2018, 9% in 2019, 17% in 2020, 16% in 2021, and 23% in 2022. A similar trend was observed for women senior authorship (P < 0.0001) (6% in 2018, 9% in 2019, 14% in 2020, 15% in 2021, and 18% in 2022) as well as for editorials with all-women authorship (P < 0.0001), increasing steadily from 9% in 2018 to 20% in 2022.

**Conclusion:**

Women authorship in editorials published in cardiothoracic surgery journals has steadily increased in recent years. Despite progress, women still make up less than a quarter of first and senior authors, highlighting a critical gap in gender equity in academic leadership that must be urgently addressed.

## INTRODUCTION

**Table:** 

Abbreviations, Acronyms & Symbols	
AAMC	= Association of American Medical Colleges
AATS	= American Association for Thoracic Surgery
CI	= Confidence interval
OR	= Odds ratio
SE	= Standard error
STS	= Society of Thoracic Surgeons

Gender disparities in cardiothoracic surgery are well-documented, with women significantly being underrepresented in the field. A recent global quantification of women cardiac surgeons reported that women constitute 8.0% of the international cardiac surgical workforce, with North America and Europe leading regional representation^[1]^. Despite increasing numbers of women entering medical school and surgical training, their representation in academic leadership, research, and faculty positions remains disproportionately low. This impacts career progression for women and reduces mentorship opportunities and role models for junior trainees^[1]^. The proportion of women residents starts higher and then narrows when becoming board-certified cardiothoracic surgeons, and most women surgeons maintain positions as assistant or associate professors rather than full professors and department chairs^[2-4]^. Relative to academic performance, women constitute a small proportion of authors on published academic articles and fulfill a minority of leading roles at major scientific conferences^[5,6]^. Peer reviewed publications are often seen as metrics of academic success, and invited editorials represent the recognition of expertise of the invited physician in the cardiothoracic surgery field. Women's representation in literature continues to lag behind that of men. Women authors are cited less often than men counterparts, publish fewer articles, and occupy fewer editorial board positions in comparison to their counterparts who are men^[7,8]^. The landscape of women authorship is, however, evolving.

Sex and gender are distinct but interconnected concepts that are crucial to consider when analyzing representation in cardiothoracic surgery. For the purposes of this paper, the distinction can be made that sex refers to biological characteristics such as chromosomes, hormones, and reproductive anatomy, while gender is a social construct that encompasses roles, behaviors, and identities shaped by cultural and societal norms^[9]^. In medical and academic contexts, differentiating between the two is essential, as gender-based disparities often result from systemic biases and structural barriers, which we aim to highlight in this study^[10]^. We recognize that this distinction ensures a more nuanced understanding of women’s representation in cardiothoracic surgery journals and helps address sociocultural challenges.

In this study, we sought to identify the current trends in gender disparity amongst authors of invited editorials published in cardiothoracic surgery journals across the globe.

## METHODS

### Search Strategy

We performed an analysis of editorials published across 16 cardiothoracic surgery journals from January 2018 to October 2022. Eligible journals were identified using the online list available at https://www.ctsnet.org/additional-journals. Additional manual search in Google was also performed to capture any additional journals. Websites of the identified journals were manually searched for articles classified specifically as editorials. Articles classified as invited commentaries, letters to the editor, review articles, and other forms of invited articles that were not explicitly deemed editorials by the journal were excluded from this review. The impact factors of all journals included were determined from the Journal Citation Reports database (Clarivate Analytics).

### Data Extraction

Upon identifying those articles classified as an editorial from each of the journals of interest, all variables were manually harvested from each article assessed. In addition to the title of study, year of study, and country of study, these variables included: total number of women-identifying authors, total number of first authors who identify as woman, and number of senior authors that identify as woman.

### Author Gender Identification

The author’s gender was predicted using the validated database Gender API (https://gender-api.com/), a web-based application with a predictive algorithm that cross-matches an online database with more than 250,000 names across all countries. Gender API classified the names of first and last authors as men, women, or unknown. Gender was assigned if Gender API predicted the gender of the author at a probability of ≥ 90%. Additionally, if there was uncertainty regarding the gender of an author, a manual review of the author’s institutional websites was completed, if available and accessible.

### Statistical Analysis

The proportion of women first authors, senior authors, and total number of women authors were calculated for each journal and year. Categorical variables were compared using the x2 test of independence. Cochran-Armitage tests were run to compare the trends in the proportion of all women authors, woman first authors, and woman senior authors across the years. Linear regressions were used to assess the association between year and the number of women authors, woman first authors, and woman senior authors. Logistic regressions were performed to calculate the association between having a woman first author and a woman senior author, and the associations between woman first and senior author positions and time, as well as the total number of women authors. Statistical analyses were performed using RStudio software, version 2023.09.1+494 with statistical significance set at a *P*-value of 0.05.

## RESULTS

### Gender Differences

A total of 16 cardiac surgery journals were assessed, representing 806 editorials published between 2018 and 2022 (Tables 1 and 2). The journal impact factors ranged from 0.3 to 6.5. The number of editorials ranged from one to 191, and there was a mean of 50.4 ± 47.6 articles per journal. Of the 1,858 article authors, 293 (16%) were women. In terms of first authors, 14% were women (n = 113) compared to women comprising 12% of senior authors (n = 94). Thoracic Surgery Clinics had the greatest percentage of total women authors (45%). Table 3 compares women editorial authorship during the study period by journal.

**Table 1:** Characteristics of included journals.

Journal name	Geographic region	2018	2019	2020	2021	2022
Annals of Cardiothoracic Surgery	China	2.9	3.1	4.1	4.6	3.1
Asian Cardiovascular and Thoracic Annals	England	-	-	-	-	0.7
Brazilian Journal of Cardiovascular Surgery	Brazil	0.8	1.5	1.3	1.3	1.3
Cirugia Cardiovascular	Spain	-	-	-	-	0.3
General Thoracic and Cardiovascular Surgery	Japan	1.2	1.1	1.5	1.2	1.2
Indian Journal of Thoracic and Cardiovascular Surgery	India	-	-	-	-	0.7
Innovations: Technology and Techniques in Cardiothoracic and Vascular Surgery	United States of America	-	-	-	-	1.5
Interactive Cardiovascular and Thoracic Surgery	England	1.9	1.7	1.9	2.0	1.7
Journal of Cardiac Surgery	United States of America	-	1.5	1.6	1.8	1.6
Portuguese Journal of Cardiac Thoracic and Vascular Surgery	Portugal	-	-	-	-	-
Seminars in Thoracic and Cardiovascular Surgery	United States of America	-	2.1	2.0	2.4	2.5
Annals of Thoracic Surgery	United States of America	3.9	3.6	4.3	5.1	4.6
The Journal of Thoracic and Cardiovascular Surgery	United States of America	5.3	4.5	5.2	6.5	6
Thoracic and Cardiovascular Surgeon	Germany	1.2	1.2	1.8	1.8	1.5
Thoracic Surgery Clinics	United States of America	1.8	1.3	1.8	3.0	2.1
World Journal for Pediatric and Congenital Heart Surgery	United States of America	-	-	-	-	0.9

**Table 2:** Number of editorials between 2018 and 2022 by journal.

Journal name	2018	2019	2020	2021	2022	Total editorials
Annals of Cardiothoracic Surgery	0	19	34	30	19	102
Asian Cardiovascular and Thoracic Annals	0	1	3	4	2	10
Brazilian Journal of Cardiovascular Surgery	14	15	20	7	5	61
Cirugia Cardiovascular	5	10	8	12	10	45
General Thoracic and Cardiovascular Surgery	0	0	0	0	1	1
Indian Journal of Thoracic and Cardiovascular Surgery	13	13	16	17	11	70
Innovations	1	5	25	17	8	56
Interactive Cardiovascular and Thoracic Surgery	4	1	2	1	6	14
Journal of Cardiac Surgery	1	4	18	22	10	55
Portuguese Journal of Cardiac Thoracic and Vascular Surgery	0	16	14	16	8	54
Seminars in Thoracic and Cardiovascular Surgery	0	1	0	0	0	1
The Annals of Thoracic Surgery	11	14	19	15	15	74
The Journal of Thoracic and Cardiovascular Surgery	162	22	2	3	2	191
The Thoracic and Cardiovascular Surgeon	8	12	8	8	5	41
Thoracic Surgery Clinics	2	3	4	6	6	21
World Journal for Pediatric and Congenital Heart Surgery	3	2	2	1	2	10
Totals	224	138	175	159	110	806

**Table 3:** Number of women first authors, senior authors, and co-authors by journal.

Journal	Total editorials	Women first authors (% of journal)	Women senior authors (% of journal)	Total authors	Number of women authors (% of journal)
Annals of Cardiothoracic Surgery	102	19 (19)	12 (12)	314	60 (19)
Asian Cardiovascular and Thoracic Annals	10	1 (10)	0 (0)	17	1 (6)
Brazilian Journal of Cardiovascular Surgery	61	3 (5)	7 (12)	146	17 (12)
Cirugia Cardiovascular	45	4 (9)	5 (11)	88	13 (15)
General Thoracic and Cardiovascular Surgery	1	0 (0)	0 (0)	3	0 (0)
Indian Journal of Thoracic and Cardiovascular Surgery	70	1 (1)	1 (1)	82	2 (2)
Innovations: Technology and Techniques in Cardiothoracic and Vascular Surgery	56	11 (20)	7 (13)	166	23 (14)
Interactive Cardiovascular and Thoracic Surgery	14	2 (14)	3 (21)	95	13 (14)
Journal of Cardiac Surgery	55	9 (16)	3 (6)	202	27 (14)
Portuguese Journal of Cardiac Thoracic and Vascular Surgery	54	22 (41)	20 (37)	72	31 (43)
Seminars in Thoracic and Cardiovascular Surgery	1	0 (0)	0 (0)	3	0 (12)
The Annals of Thoracic Surgery	74	15 (20)	14 (19)	266	59 (22)
The Journal of Thoracic and Cardiovascular Surgery	191	17 (9)	11 (6)	320	32 (10)
The Thoracic and Cardiovascular Surgeon	41	0 (0)	0 (0)	41	0 (0)
Thoracic Surgery Clinics	21	11 (52)	11 (52)	29	13 (45)
World Journal for Pediatric and Congenital Heart Surgery	10	0 (0)	0 (0)	14	0 (0)

### Regional Trends

**Fig. 1:**
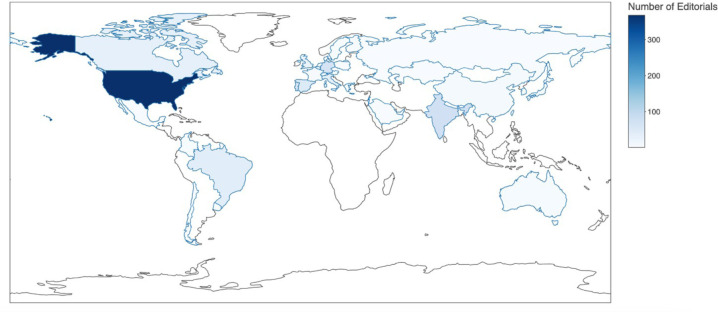
Heat map of regional trends of included editorials.

Most editorials were published by authors from the United States of America (45%) followed by India (9%) and Germany (8%) (Table 4). The United States of America was the country with the highest number of women as authors. Proportionally, 17% of articles from the United States of America had women as first authors, and 14% had women as senior authors. In India, there was one woman first author and one woman senior author, each representing 1%, and Germany had no editorials with women as first authors or senior authors. Figure 1 provides a visual summary of global trends of included editorials.

**Table 4:** Trends in editorial authorship across countries of included journals.

Country	Total editorials	Women first authors (% of country)	Women senior authors (% of country)	Total authors	Women authors (% of country)
Australia	4	0 (0)	0 (0)	7	0 (0)
Austria	2	0 (0)	0 (0)	8	2 (25)
Belgium	7	2 (2)	1 (14)	46	4 (9)
Brazil	42	1 (2)	3 (7)	103	9 (9)
Canada	31	6 (19)	2 (7)	81	15 (19)
Chile	1	0 (0)	0 (0)	1	0 (0)
China	6	0 (0)	0 (0)	17	0 (0)
Colombia	2	0 (0)	1 (50)	7	2 (29)
Denmark	1	1 (100)	0 (0)	3	1 (33)
Finland	3	1 (33)	2 (67)	7	3 (43)
France	3	0 (0)	0 (0)	8	0 (0)
Germany	65	0 (0)	0 (0)	105	3 (3)
Greece	2	0 (0)	0 (0)	7	0 (0)
Hong Kong	1	1 (100)	0 (0)	5	2 (40)
India	72	1 (1)	1 (1)	92	2 (3)
Israel	3	0 (0)	0 (0)	6	0 (0)
Italy	34	8 (24)	2 (6)	139	33 (24)
Japan	8	1 (13)	0 (0)	14	1 (7)
Kazakhstan	1	0 (0)	1 (100)	2	1 (50)
Mexico	1	0 (0)	0 (0)	1	0 (0)
Netherlands	7	2 (29)	2 (29)	67	10 (15)
Poland	1	1 (100)	0 (0)	6	1 (17)
Portugal	56	22 (40)	20 (36)	74	31 (42)
Russian Federation	1	0 (0)	0 (0)	5	0 (0)
Saudi Arabia	1	0 (0)	0 (0)	4	2 (50)
South Korea	1	0 (0)	0 (0)	2	0 (0)
Spain	48	5 (10)	7 (15)	94	17 (18)
Sweden	2	0 (0)	0 (0)	7	0 (0)
Switzerland	5	0 (0)	0 (0)	11	0 (0)
Taiwan	1	0 (0)	0 (0)	3	1 (33)
United Arab Emirates	1	0 (0)	0 (0)	1	0 (0)
United Kingdom	23	2 (9)	1 (4)	66	8 (12)
United States of America	368	61 (17)	51 (14)	848	143 (17)
Uruguay	2	0 (0)	0 (0)	11	0 (0)

### Temporal Trends

**Fig. 2:**
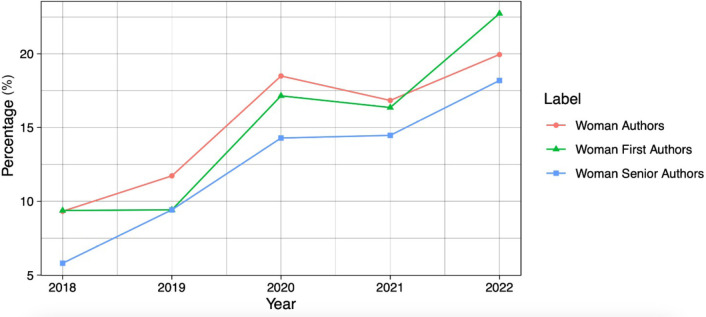
Proportion of women as authors in cardiothoracic surgery journal editorials between 2018 and 2022.

From 2018 to 2022, there was a significant increasing trend in the proportion of women as first authors (*P* < 0.001). Specifically, 9% in 2018 (n = 21), 9% in 2019 (n = 13), 17% in 2020 (n = 30), 16% in 2021 (n = 26), and 23% in 2022 (n = 23). Further, there was an increasing trend in both the proportion of women as senior authors (6% in 2018, 9% in 2019, 14% in 2020, 15% in 2021, and 18% in 2022, *P* < 0.0001) and percentage of total women authors (9% in 2018, 12% in 2019, 19% in 2020, 17% in 2021, and 20% in 2022, *P* < 0.0001) across the years (Figure 2). The linear regressions revealed that there was an average increase of 2.64-women authors per year (β = 2.6352, standard error [SE] = 0.6622, R^2^ = 0.8407, *P* = 0.0284), an average increase of 3.36 women first authors per year (β = 3.3636, SE = 0.7249, R^2^ = 0.8777, *P* = 0.0189), and an average increase of 2.98 senior authors per year (β = 3.3636, SE = 0.7249, R^2^ = 0.8777, *P* = 0.0189).

According to logistic regression analysis, each successive year significantly predicted an increase in first authors (odds ratio [OR] = 1.3, 95% confidence interval [CI] = 1.1 - 1.5, *P* < 0.001) and senior authors who were women (OR = 1.4, 95% CI = 1.2 - 1.6, *P* < 0.001). A higher number of women authors per editorial was significantly associated with having a woman first author (OR = 9.3, 95% CI = 6.5 - 13.8, *P* < 0.0001) and woman senior author (OR = 5.9, 95% CI = 4.3 - 8.3, *P* < 0.0001).

## DISCUSSION

In our analysis of editorials published in cardiothoracic surgery journals, we examined authorship trends to assess the representation of women between 2018 and 2022. We observed an increase in women’s authorship over the examined time period, though women still represented less than a quarter of editorial authors altogether (16%), and even less of first authors (14%), and senior authors (12%). As well, women’s representation in authorship positions varied greatly by journal and country.

Addressing women's underrepresentation in academic cardiothoracic surgery requires a deeper understanding of the systemic factors driving this disparity^[11]^. Contributing barriers include gender bias and sociocultural expectations^[12,13]^. For example, women are more likely than men to leave surgical residency, influenced by higher rates of burnout, depression, and suicidal ideation^[14]^; a lack of role of models and mentors^[15]^; gender discrimination^[16]^; and outdated attitudes toward family dynamics, such as biases related to childbirth and maternal roles^[15]^. Though North America and Europe are home to the largest proportion of women cardiac surgeons, other regions, especially East Asian and Middle Eastern countries, have strikingly low numbers of women cardiac surgeons due to these limitations^[14]^.

After considering systemic factors that contribute to gender disparities in cardiothoracic surgery, it is important to identify obstacles specific to professional growth in academia. Implicit biases in peer review may compromise objectivity, posing a barrier to successful publication. It has been shown that manuscripts with men as a senior author are more likely to be accepted than those led by women^[17,18]^. This disparity is particularly pronounced when reviewer panels consist solely of men. These findings suggest that women authors may face disproportionately harsh critiques unrelated to the quality of their work. Additionally, the male-dominated composition of editorial boards and reviewer pools further reinforces these inequities in publication decisions^[18]^. Addressing these biases requires greater transparency in peer review, increased diversity in editorial leadership, and policies that promote gender equity in academic publishing.

Though women’s representation in first author positions in high-impact medical journals has increased over the past few decades, this chance has unfortunately plateaued or even declined in some publications, suggesting that women are consistently underrepresented^[8,19,20]^. Compared to men, women are still less likely to be published as first authors in high-impact medical journals such as The British Medical Journal and The Lancet, even when adjusting for specialty^[20]^. Publications in high-impact journals serve as markers of research excellence and innovation, directly influencing career advancement. Similar to impact factors, editorials represent recognition of an author’s expertise, productivity, and reputation. Therefore, invitations to write editorials are typically extended to accomplished senior scholars, often through informal peer networks^[5]^. However, the results of this study demonstrated that women remain underrepresented among invited editorialists in cardiothoracic surgery journals, reflecting persistent gender inequities in academic recognition and leadership opportunities.

In scholarly publishing, the allocation of research credit is a fundamental aspect of scientific authorship, with significant implications for career advancement^[21-23]^. Qualitative research suggests that the discrepancy observed in women's representation in science may not stem from inadequate contributions but rather from a lack of recognition^[11,13]^. This systemic undervaluation may contribute to fewer women securing key authorship positions. A study evaluating women’s authorship trends across all peer-reviewed article types in two major cardiothoracic surgery journals found that women comprised only overall 16.7% of first authors and 8.1% of senior authors, highlighting that the identified trend is not exclusive to editorial articles^[5]^. Another bibliometric analysis examining gender authorship trends from 2015 to 2019 in vascular surgery journals found that women represented a quarter of all first authors and only 10% of all senior authors, with no significant increase in women last authors over time^[24]^. Our findings suggest that having a woman as the first or senior author increases women’s representation among co-authors, reinforcing the role of women-led mentorship. This dynamic enhances the productivity of women researchers, ultimately strengthening the pipeline to senior authorship and promoting long-term gender equity in academic publishing.

Membership within an editorial board is a prestigious academic position that signifies scholarship and expertise within a given discipline. Thus, scientific productivity and records of leadership in research are required for such a role. A study investigating the composition of editorial boards of cardiothoracic surgery journals identified that across 22 journals, only 10.5% of editorial board members were women, and only one journal had a woman Editor-in-Chief^[25]^. Another analysis of women’s representation among editorial boards of two high-impact cardiothoracic journals, The Annals of Thoracic Surgery and the Journal of Thoracic and Cardiovascular Surgery, found that while the proportion of women holding editorial board positions has increased at a rate similar to the growth of practicing women cardiothoracic surgeons, the overall representation of women in editorial roles (18% in 2023) remains disproportionately low compared to their presence in the field^[26]^. Similarly, in terms of gender distribution among presidential roles of cardiothoracic surgical societies globally, there has been a stark lack in the number of women occupying the role^[27]^. Without fair opportunities for women in academic cardiothoracic surgery to publish as first or senior authors, they cannot acquire the necessary recognition to advance to editorial board positions. The limited representation of women on editorial boards highlights the need to identify barriers and facilitators to their training and advancement into leadership roles in cardiothoracic surgery.

Gender-based salary disparities persist among cardiac surgeons, with women consistently earning less than their male counterparts. In 2021, cardiothoracic surgeons who are women earned between $0.71 - $0.86 for every dollar earned by surgeons who are men^[28]^. This disparity widened with higher academic ranks; notably, professors who are women earned less than their counterparts, and in some cases, even less than lower-ranked colleagues who are men. Between 2019 and 2021, while surgeons who are men at the associate professor, professor, and chief levels saw increases in mean salaries, women academic surgeons experienced decreases. Industry payments also reflect this imbalance. In 2022, male cardiothoracic surgeons who are men received 92.4% of total industry payments, amounting to $10,967,855, with a median payment of $6,611. In contrast, women received only 7.6% of these payments^[29]^. Furthermore, in the United States of America, an analysis of Medicare payments in 2019 revealed that women cardiothoracic surgeons received significantly lower mean payments than their peers who are men, even after adjusting for factors such as the number of services provided, unique billing codes, patient complexity, years in practice, and regional population density^[30]^. These findings underscore the persistent gender pay gap in cardiothoracic surgery, which may discourage women from pursuing career advancement and highlight the need for continued efforts to achieve salary equity in the field.

Overall, the results of our study are consistent with trends identified in recent literature regarding women’s representation in academic cardiothoracic surgery and broader medical research. Collectively, these findings highlight the need to improve women's representation in academic cardiothoracic surgery. When considering strategies to address these gender inequities, various efforts have been made, including the development of advocacy groups and the adjustment of financial compensation structures.

### What Is Being Done to Address This Issue?

Gender parity in surgery refers to the equal representation and participation of women and men in the surgical field, particularly in leadership roles, training programs, and professional opportunities. It is measured by the ratio of women to men in surgical specialties, academic positions, and hospital leadership. One single institution study reviewed surgery faculty salaries and revealed significant gender disparity with women faculty being compensated less than their men counterparts; implementing a university-wide objective compensation plan, the institution raised faculty salaries to match the Association of American Medical Colleges (AAMC) regional median salary, thus addressing gender-based salary inequity^[31]^. While this is not specific to the discipline of cardiothoracic surgery, implementing such plans broadly and across the field should be considered. Publishing detailed analyses of gender-based salary differences has heightened awareness within the medical community. For instance, a study utilizing data from the 2019 Society of Thoracic Surgeons (STS) Practice Survey revealed that women in cardiothoracic surgery were more likely to report lower salaries compared to their male counterparts, even when controlling for factors like age and years in practice^[32]^.

Implementing policies that promote gender equity, such as transparent pay structures and equitable hiring practices, is crucial. The AAMC, for example, has developed standards to promote a culture that enhances female recruitment and encourages a strong network of women faculty^[33]^.

Establishing mentorship initiatives aims to support female surgeons' career advancement. Increasing access to mentors, promoting positive behavior, and minimizing detrimental behavior may enhance support for female cardiothoracic surgeons^[34]^. Moreover, the scarcity of women in faculty positions may contribute to the early discouragement of junior women researchers^[35]^. Notably, over the past five years, the steady rise in women’s editorial authorship can be viewed as an indicator of growing mentorship in the field^[36]^. In a recent commentary published in the Journal of Thoracic and Cardiovascular Surgery, authors highlight that crossing the gap between first and last author does not happen without guidance or mentorship^[36]^. A study looking at 5,000 mentor-mentee pairs found that individuals with publications co-authored with leading scientists in their fields were more likely to achieve academic success^[37]^.

The STS and the American Association for Thoracic Surgery (AATS) have taken several initiatives to promote women’s authorship and address gender disparities in academic publishing within the field of cardiothoracic surgery^[38]^. In particular, both organizations support the Women in Thoracic Surgery initiative that aims to promote professional development and advancement of women in cardiothoracic surgery, by providing networking opportunities and mentoring programs to support women surgeons in their careers^[39]^. In recognizing that a majority of cardiothoracic surgery trainees in the United States of America have reported gaps in terms of career path guidance, assistance obtaining a job, and advice regarding work-life balance, AATS has developed a series of educational programs and workshops that are focused on topics relevant to women in cardiothoracic surgery, including leadership skills, work-life balance, and overcoming barriers to advancement^[38-40]^. Specifically, AATS has developed a Women in Cardiothoracic Leadership webinar series that provides the opportunity for attendees to learn from and engage with cardiothoracic women surgical experts^[38]^. These webinars are focused on recruitment of women to cardiothoracic surgery at the level of high school, undergraduate, medical school, and residency trainees, with a goal of improving access to mentorship opportunities and providing transparency on the specialty^[38]^. Finally, both societies are dedicated to the promotion of women in leadership positions within their organizations, including encouraging women to serve on committees, task forces, and the Board of Directors, providing them opportunities to influence decision-making and inspire the next generation of surgeons with mentorship opportunities^[38,39]^. Additionally, groups such as the Gender Equity Initiative in Global Surgery work to address gender disparities through research, advocacy, and mentorship, striving for worldwide gender equity in surgery by 2030^[41]^.

### Limitations

There are a few important limitations to the present study. First, binary classification was used to report author gender, and data extraction involved stating either “yes” or “no” to indicate whether the author is a woman. However, self-identified gender may vary from the author’s assumed binary identification, and dichotomization of authors as men or women can introduce ethical concerns^[42,43]^. Second, Gender API was used to determine the author’s gender from their first name and country of origin. Despite being more reliable than comparable software^[6]^, Gender API is still imperfect and subject to error. Confirmation through online searches did not always guarantee accurate gender identification, particularly for women with gender-neutral, less conventionally feminine, or exceptionally uncommon names, potentially leading to an underestimation of total women authors. Also, we did not internally validate Gender API’s performance and thus cannot report an error rate. However, our selection was based on prior literature citing its reliability, efficiency, and accuracy^[43-45]^. Gender API supports multiple languages and handles large databases, making it a strong choice for gender detection in research. Its proven performance has established it as a preferred tool in this field.

## CONCLUSION

Our findings show that women’s representation in editorial authorship in cardiothoracic surgical journals has steadily increased over the past several years, however, it has remained significantly lower compared to men. Advocating for gender equity and actively providing support to women surgeons, their academic career will likely need to remain key priorities for medical institutions, professional associations, and current academic leaders in cardiothoracic surgery in order to close the identified gaps in this study. Our results shed light on the current state of gender disparities in academic cardiothoracic surgery and should be used to inform relevant initiatives and future research directions that work to eliminate the gender gap.

## Data Availability

The authors declare that the data supporting the findings of this study are available within the article.

## Artificial Intelligence Usage

The authors declare that no artificial intelligence tool was used in the preparation of this article.
